# Improving reproducibility in spine research

**DOI:** 10.1002/jsp2.1127

**Published:** 2020-09-29

**Authors:** Chitra L. Dahia, Christine L. Le Maitre

**Affiliations:** ^1^ Department of Cell and Developmental Biology Weill Cornell Medicine, Graduate School of Medical Sciences New York New York USA; ^2^ Hospital for Special Surgery New York New York USA; ^3^ Biomolecular Sciences Research Centre Sheffield Hallam University Sheffield UK

Back pain is the leading cause of disability worldwide, with incidence increasing with the aging population. However, therapies for back pain have shown little improvement over the last 20 years, although there are a number of exciting avenues under investigation. Back pain is a heterogeneous condition with multifactorial causes, although around 40% of chronic back pain cases have been linked to degeneration of the intervertebral disc, there are no therapies in widespread use which target the degenerate disc per se. Researchers worldwide studying spinal conditions are multi‐disciplinary ranging from developmental biologists, cell and molecular biologists, tissue engineers, biomechanics, radiologists, neurologists, and musculoskeletal clinicians using various preclinical animal models ranging from zebrafish, chicken, rodents, bovine, porcine, ovine, and primates to those which utilize human tissues and cells from surgical samples and cadavers. Many studies require a cross‐disciplinary approach to test hypotheses and to enable acceleration of research there is an urgent need for more collaborative research approaches.

The reproducibility of scientific methods is critical for forming a platform for future studies. Reproducibility is crucial by independently repeated experiments, and more robustly when repeated by another lab. The technical details on methodology, reagents, equipment, and well characterized protocols are very important for reproducibility of scientific findings. These details on specific cell or tissue type and model systems are also useful for a researcher starting a new technique within the lab or clinical research, for successful adaptation of methods, and advancement of science in a time and resource efficient manner. Hence, this Special Issue was developed to solicit and share methods and protocols for the research in spine and intervertebral disc.

In this Special Issue on *The Methods, Protocols and Resources in Spine Research*, we have assembled papers covering the major approaches used for spine and disc research. While a particular method may be published using a given experimental system, sufficient details are provided for adaption by various model systems. Papers published within this issue provide essential guidance and methodology which can be applied across the spectrum of research specialisms, including a number of collaboration papers where consensus methods have been developed.

## 1 CELL, MOLECULAR, AND DEVELOPMENTAL BIOLOGY

- Binch et al^1^ shared the principles and protocols on immunostaining of the intervertebral disc, together with hints and tips on how to optimize any antibody for the intervertebral disc, with advice on what to avoid. Immunohistochemistry is a versatile technique enabling the detection and localization of specific protein expression, which is utilized in studies including developmental, pathogenesis, and investigating outcome measures for therapeutic approaches.
- Piprode et al^2^ shared two protocols: one for isolation of each component of the murine intervertebral disc including nucleus pulposus, annulus fibrosus, and endplate cells, that can be used for further characterization by high‐throughput omics approaches including single cell RNAseq; and a protocol for isolation for high‐quality RNA along with pointers to simplify adaption. These methods will be useful for the rigorous analysis of IVD cells and will aid in the understanding of intervertebral disc biology. While these methods were designed for mouse models they can also be adapted for other species within the community.
- Veras et al^3^ reports a collaborative development of a consensus methodology for the parallel proteomic and metabolomic profiling of mouse intervertebral disc. The methodology covered describes the dissection, extraction of cells specifically for omics applications and data analysis, providing considerations for the small starting materials and successful extraction from the problematic ECM rich tissues of the IVD. This paper also reported methods for mouse tissues which can be adapted for other species.

## 2 BIOMECHANICS

- Newell et al^4^ report on the repeatability of compressive loading systems on bovine motion segments across three laboratories, providing standardized test protocols for comparison of spinal segments. Of interest, they noted differences in animal samples between the United States and United Kingdom suggesting potential mechanical differences between bovine breeds or maintenance conditions of the animals.
- Shekouhi et al^5^ shared an in silico approach for evaluating the spinal implants of growing rods used for scoliosis correction surgeries. This in silico model enables the simulation of clinical parameters which have not been previously possible.
- Takeoda et al^6^ developed and characterized a novel cell culture system using a semipermeable membrane pouch device to test the effects of culturing bovine nucleus pulposus cells under constant and cyclic hydrostatic pressure under high osmolality. This culture system and pouches have potential use for testing the effects of motion on biochemical changes in the nucleus pulposus cells in vitro.
- Di Pauli et al^7^ compare five different methods to determine neutral zone of rat spinal motion segments using two different load deflection profiles demonstrating poor agreement between methodologies, highlighting the need for further work to identify recommended methods which are most sensitive to disc degeneration and can be used in treatment studies.

## 3 PRECLINICAL MODELS

- Du et al^8^ report the development of an organ culture system of bovine intervertebral discs in the presence of TNF‐ɑ to test the effects of anti‐inflammatory treatment, together with translation to human IVD cells.
- Glaeser et al^9^ utilized a needle‐puncture induced rat lumbar disc degeneration model to develop a reproducible and standardized small animal model of painful IVD degeneration that will be useful to assess the functional outcome of pain. Pain assessments in animal models have been a key limitation for developing disc therapies.
- Lin et al^10^ compared six commercially available cellular bone matrices using athymic rat model of posterolateral fusion at the bone quality and efficiency of spinal fusion enabling the direct comparison of fusion systems within a controlled animal model.

## 4 IMAGING

- Pai et al^11^ provide important methodology for the determination of muscle morphology in the thoracic spine using MRI‐based measurements; these methods can be utilized to enable comparisons of muscle morphometry across different studies globally and will aid the understanding of the roles of muscle morphology in spinal physiology and pathology.
- Cauble et al^12^ detailed the characterization of Atomic Force Microscopy to quantify the extracellular matrix structure, at submicron size using skeletally mature murine model of needle‐puncture induced progressive caudal IVD degeneration. This method quantifies the Collagen‐D spacing and can quantify changes in both nucleus pulposus and annulus fibrosus of the discs. While this method was characterized using a murine model, it can be adapted to all preclinical animal models and will be instrumental in quantifying microstructural changes during disc progression or for testing therapeutics.
- Grindle et al^13^ contributed their protocol on radiological measurements of spinal kyphosis and compared the outcome of standing radiograph measurements with flexicurve and motion analysis markers. These methodologies enable improved understanding of in vivo biomechanical contribution to spinal conditions.
- Meadows et al^14^ present methodology for developing reproducible T2 measurements from MRI data utilizing curve fitting methods by correcting for Rician noise. These methods will be particularly useful for determination of T2 measurements in degenerated discs and the AF where the signal‐to‐noise ratio is lower.

Future issues in *JOR Spine* will report on consensus methodology which is currently under development by international collaborations across the spinal community, including consensus on nomenclature, histological grading, and in vitro culture of IVD cells.

## CONFLICT OF INTEREST

The authors declared no potential conflicts of interest.

## AUTHOR CONTRIBUTIONS

Chitra L. Dahia and Christine L. Le Maitre contributed equally to conceptualization, writing original draft, and finalizing the editorial.
